# A Neonatal Case of Paraesophageal Mixed Hiatal Hernia Detected by “Coiled-Up Sign”

**DOI:** 10.70352/scrj.cr.25-0461

**Published:** 2025-10-25

**Authors:** Yoshiki Yamaguchi, Mutsumi Nakamura, Akihiko Tamaki, Ryuichiro Hirose, Makoto Hayashida

**Affiliations:** 1 Department of Pediatric Surgery, Fukuoka Children’s Hospital, Fukuoka, Fukuoka, Japan; 2 Department of Thoracic, Breast and Pediatric Surgery, Fukuoka University Hospital, Fukuoka, Fukuoka, Japan; 3 Chiisana Clinic, Fukuoka, Fukuoka, Japan

**Keywords:** neonate, paraesophageal mixed hiatal hernia, coiled-up sign, laparoscopic surgery, fundoplication

## Abstract

**INTRODUCTION:**

Paraesophageal mixed hiatal hernia is a rare entity in neonates, that occasionally induces near-complete esophageal or gastric obstruction, and its diagnosis is sometimes difficult. Furthermore, its management remains controversial. This case provides new insights into the diagnosis and treatment strategies of neonatal paraesophageal mixed hiatal hernias.

**CASE PRESENTATION:**

The case was a 4-day-old female infant who had frequent emesis after feeding. Plain radiography showed a “coiled-up sign” of an orogastric tube at the level of the diaphragm. CT indicated that the upper half of the stomach protruded into the mediastinal space. In the upper gastrointestinal study, the abdominal esophagus and cardia were compressed and bent, causing the contrast material to stagnate. Thus, she was diagnosed with paraesophageal mixed hiatal hernia. The enteral tube was advanced to the level of the jejunum by insufflating the stomach and returning it to the abdominal cavity under fluoroscopy. After feeding via an enteral tube during the neonatal period, the patient underwent radical surgery at 37 days old. We performed laparoscopic procedures involving pulling the stomach down, approximating the widened hiatus, and Toupet fundoplication to prevent postoperative gastroesophageal reflux.

**CONCLUSIONS:**

The “coiled-up sign” of the gastric tube at the level of the diaphragm in neonates suspected with upper gastrointestinal obstruction or stenosis should raise suspicion of a paraesophageal mixed hiatal hernia and an upper gastrointestinal contrast study is useful for the diagnosis. Insertion of a feeding tube may allow for elective radical surgery while avoiding life-threatening complications.

## Abbreviation

EGJ
: esophagogastric junction

## INTRODUCTION

Among congenital esophageal hiatal hernias recognized in the neonatal or infantile period, extensive prolapse of the stomach is rarely observed. Hiatal hernia in which a large portion of the stomach (>30%) herniates into the mediastinal space is called “giant hiatal hernia”^1)^ and is classified as type III, paraesophageal mixed hiatal hernia. Most cases of paraesophageal hernia in neonates and infants are included in this type^2)^ and may show different clinical manifestations from sliding hiatal hernia, in which the pathology is mainly due to gastroesophageal reflux. A largely prolapsed stomach may compress and bend the abdominal esophagus and cardia, causing severe obstruction. In addition, it may also lose fixation and be prone to volvulus, leading to perforation.^3)^ Definitive diagnosis in neonates and infants is occasionally difficult, and some cases require emergency surgeries due to these threatening complications.^4)^ However, the procedures in the neonatal period are challenging.

We herein report the neonatal case of a paraesophageal mixed hiatal hernia, whose diagnosis was triggered by plain radiography. This case provides new insights into the diagnosis and treatment strategies of neonatal paraesophageal mixed hiatal hernias.

## CASE PRESENTATION

A 4-day-old female patient was referred to our institution because of post-feeding vomiting. She was born at 40 weeks and 0 days via spontaneous vaginal delivery without any complications. Her birth weight was 3080 g with Apgar scores of 8 and 9 at 1 and 5 min, respectively. Frequent brownish vomiting was observed after feeding.

The neonate was 49.6 cm tall and weighed 2762 g at the time of hospitalization. Her abdomen was mildly distended. Ultrasonography revealed left hydronephrosis; however, no other abnormalities were detected.

**Fig. 1:**
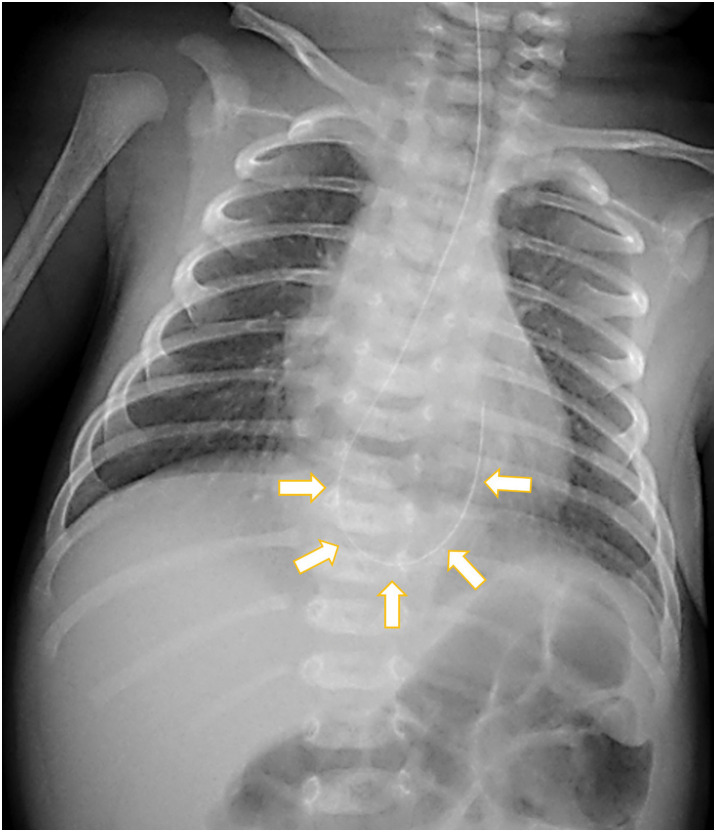
Chest radiograph. The orogastric tube curled at the level of diaphragm, which is mimicking so-called “coiled-up sign” (arrows).

**Fig. 2:**
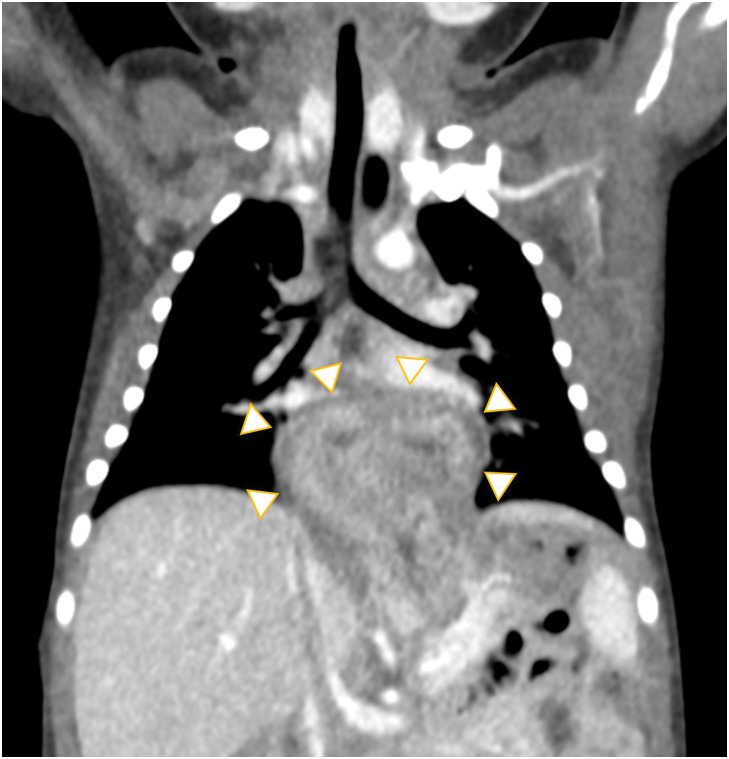
CT. The upper half of the stomach protruded into the inferior mediastinum, appearing as a dome-shaped mass (arrowheads).

**Fig. 3:**
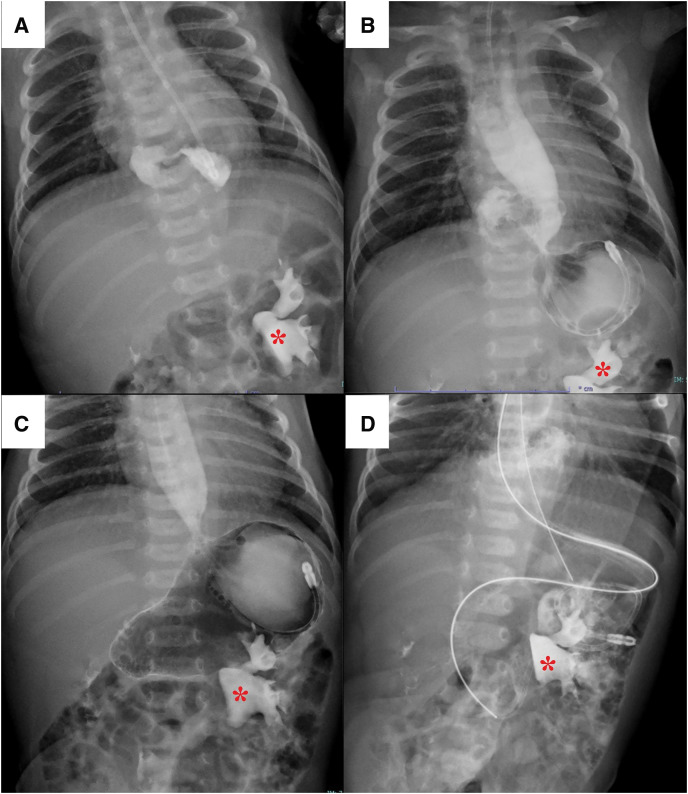
Upper gastrointestinal contrast study. Left renal pelvis distended due to hydronephrosis: contrast medium remained after the contrast CT (*). (**A**) The abdominal esophagus and cardia were compressed and bent, causing the contrast material to stagnate. (**B**) When air was insufflated into the stomach, the stomach in the abdominal cavity was observed. (**C**) As the stomach expanded, the prolapsed portion returned to the abdominal cavity. After hernia reduction, the stomach had a normal morphology. (**D**) A 5 Fr. enteral tube was placed at the level of the jejunum beyond the ligament of Treitz.

An orogastric tube was inserted 22 cm from the gum margin, but the insertion was not smooth. A chest plain radiography demonstrated that the tube curled at the level of the diaphragm, which is mimicking so-called “coiled-up sign” and indicated esophageal obstruction or stenosis (**Fig. 1**). CT showed that the upper half of the stomach protruded into the inferior mediastinum, appearing as a dome-shaped mass (**Fig. 2**). An upper gastrointestinal study revealed compression and bending of the abdominal esophagus and cardia, which caused the contrast material to stagnate (**Fig. 3A**). Based on these findings, the diagnosis was type-III paraesophageal hiatal hernia, also called mixed hiatal hernia. The esophagus was not short but dilated. Placement of an enteral tube seemed to be difficult because the protruding stomach was bent at a sharp angle. The stomach in the abdominal cavity was observed when air was insufflated into it (**Fig. 3B**). As the stomach expanded, the prolapsed portion returned to the abdominal cavity. After hernia reduction, the stomach showed a normal morphology (**Fig. 3C**). Therefore, a 5 Fr. enteral tube was placed at the level of the jejunum beyond the ligament of Treitz (**Fig. 3D**). Nutrition was provided via the tube, and the patient’s weight increased steadily. No symptoms suggestive of the acute gastric volvulus were noted during admission.

**Fig. 4:**
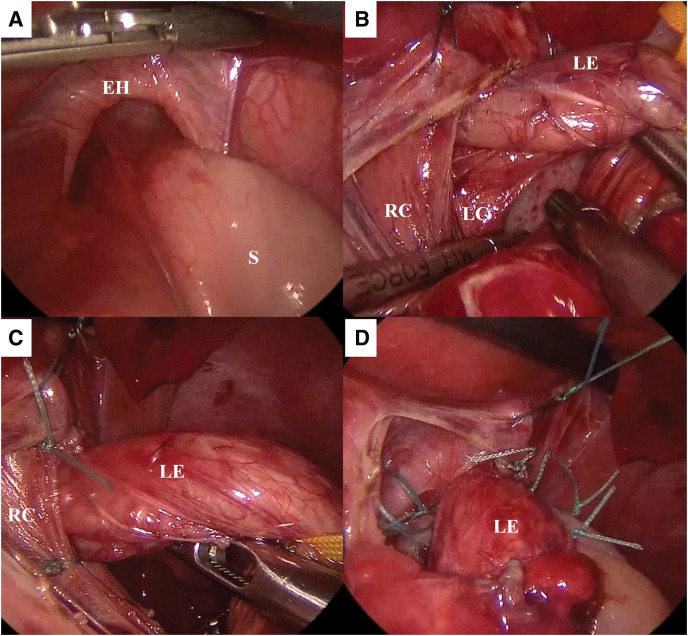
Intraoperative findings. (**A**) The esophageal hiatus was widely open about 2 cm, through which the fundus was protruded into the mediastinal space. (**B**) The bilateral crura and the lower esophagus were exposed by dissecting the phrenoesophageal membrane. (**C**) The esophageal hiatus was sutured with non-absorbable sutures on the ventral and dorsal sides of esophagus. (**D**) 180-degree Toupet fundoplication was performed using non-absorbable sutures. EH, esophageal hiatus; LC, left crus; LE, lower esophagus; RC, right crus; S, stomach

Radical laparoscopic repair was performed at 37 days old and her weight was 3780 g. A 5-mm port for the telescope was inserted through the umbilicus. Four additional ports were placed; a 5-mm port at the left upper abdomen for the surgeon’s right-hand, a 3.5-mm port at the right upper abdomen for the surgeon’s left-hand, a 5-mm port at the left subcostal anterior axillary line for the assistant, and a 5-mm port at the right subcostal anterior axillary line for support of a suture-based liver retraction. Under laparoscopic visualization, it was confirmed that the esophageal hiatus was widely open about 2 cm, through which the fundus protruded into the mediastinal space (**Fig. 4A**). The fundus could be easily pulled down. The phrenoesophageal membrane was then dissected to expose the bilateral crura and lower esophagus (**Fig. 4B**). Two short gastric vessels were divided to mobilize the fundus. The esophageal hiatus was sutured using non-absorbable sutures on the ventral and dorsal sides of the esophagus (**Fig. 4C**). Thereafter, 180° Toupet fundoplication was performed using non-absorbable sutures (**Fig. 4D**). The intraoperative blood loss was minimal, and the operative time was 243 min.

The postoperative course was uneventful. Oral feeding was initiated on POD 4. She achieved complete oral feeding without emesis and was discharged home on POD 13.

## DISCUSSION

Hiatal hernia is defined as the protrusion of the abdominal viscera, including the stomach, through the diaphragmatic hiatus into the mediastinal space.^5)^ It is divided into four classes based on the hernia contents and position of the EGJ.^6)^ In type I, a sliding hernia, the EGJ is located in the thorax. In type II, the EGJ remains below the diaphragm, whereas the gastric fundus herniates into mediastinal space. Type III has characteristics of both types I and II, with both the EGJ and gastric fundus positioned in the intrathoracic cavity. In type IV, organs other than except the stomach herniate. Types II–IV are collectively referred to as paraesophageal hernias. Paraesophageal hiatal hernias are extremely rare in neonates,^4)^ and Petrosyan et al.^2)^ reported that more than half of them were classified as type III, paraesophageal mixed hiatal hernias.

Hiatal hernias exhibit various symptoms. Patients with type I sliding hernias usually exhibit vomiting, respiratory infections, anemia, and failure to thrive due to gastroesophageal reflux.^5,7)^ Lethal complications have also been reported, such as obstruction, gastric volvulus, bleeding, and perforation in the mixed paraesophageal hernia.^3,8)^ However, we consider that complications may differ according to the degree of gastric prolapse even in the same mixed paraesophageal hernia. In hiatal hernia, the phrenoesophageal ligament surrounding the lower esophagus and cardia forms a hernia sac, determining the extent of prolapse. Many previous reports have shown large mediastinal masses on chest X-rays or dilated stomach prolapsing into the mediastinal space on upper gastrointestinal studies.^1,6–8)^ These findings indicate that the hernia sac is loose, and the protruding stomach loses its fixation and is prone to volvulus formation. Conversely, when the hernia sac is tight, as in our case, the lower esophagus and cardia are compressed and bent by the prolapsed stomach, causing obstruction. This appears to be a disease type that has not been reported before.

A hiatal hernia can be suspected when a chest radiograph shows a gastric shadow located in the lower lung field or behind the cardiac shadow,^9)^ but it is often difficult to detect. In our case, the insertion of orogastric tube was difficult and the tube got stuck. The tube curled at the level of the diaphragm on chest X-ray; this radiographic finding closely resembled the “coiled-up sign”, that indicated a blind end of the upper esophagus in the congenital esophageal atresia.^10)^ As the gastric tube was arrested at the level of the diaphragm in this case, we suspected lower esophageal obstruction or stenosis. The upper gastrointestinal contrast study showed that the abdominal esophagus and cardia were compressed and bent by the protruding upper half of the stomach, which made the gastric tube tortuous. Thus, coiling of the gastric tube on neonatal chest radiograph can indicate not only congenital esophageal atresia or stenosis but also paraesophageal mixed hiatal hernia with upper gastrointestinal obstruction.

As the present patient showed a stable general condition, we opted for elective surgery after allowing her to grow with tube feeding. Several reports recommend prompt surgery to prevent life-threatening conditions due to acute gastric volvulus, massive bleeding, or gastrointestinal perforation, even in stable patients.^3,4,7)^ However, the narrow intraabdominal space and vulnerable tissues of newborns make this challenging. Insertion of the enteral tube is occasionally difficult because of the bent stomach. Air insufflation into the stomach induced reduction of the prolapsed stomach into the abdominal cavity and facilitated tube placement. The indwelling tube seemed to contribute to the prevention of gastric bending or volvulus and the patient showed good weight gain without any problems.

The surgical procedures consisted of reduction of the hernia contents, resection of the hernial sac, and reapproximation of the hiatus.^5)^ Recently, minimally invasive surgery has been established in neonates and infants with development of laparoscopic instruments.^6)^ We also performed laparoscopic hiatal hernia repair without complications. Whether or not an anti-reflux procedure should be performed is controversial. The guideline published by the Society of American Gastrointestinal and Endoscopic Surgeons (SAGES)^11)^ recommends fundoplication for adult cases of paraesophageal hiatal hernia in terms of gastroesophageal reflux, hernia recurrence, leakage, and the quality of life. However, there is no consensus regarding the effects of fundoplication on the hiatal hernia recurrence in pediatric patients.^5,12)^ A neonatal case was reported in which the fundoplication could not be performed due to microgastria.^7)^ Conversely, Karpelowsky et al.^12)^ reported that 12 of 20 patients who did not undergo fundoplication had recurrent symptoms of reflux, compared with 6 of 39 patients who underwent fundoplication. We suspected that fundoplication might contribute to the prevention of postoperative reflux because congenital hiatal hernia is associated with congenital laxity of the gastric ligaments. In addition, a meta-analysis from 2023 by Li et al.^13)^ concluded that Toupet fundoplication was associated with lower prevalence of long-term complications like postoperative dysphagia and postoperative inability to belch than Nissen fundoplication in patients with gastroesophageal reflux disease. Analatos et al.^14)^ also reported that the Toupet fundoplication had a lower Dakkak dysphagia score than the Nissen fundoplication in patients who underwent the surgical repair of the paraesophageal hernias. As this patient had no underlying diseases other than the paraesophageal hiatal hernia, oral intake was expected to be established postoperatively. Therefore, we chose to perform Toupet fundoplication.

## CONCLUSIONS

We encountered a case of neonatal paraesophageal mixed hiatal hernia that underwent elective surgery after feeding via an enteral tube. The “coiled-up sign” of the gastric tube at the level of the diaphragm was suspected to indicate paraesophageal mixed hiatal hernia, and the diagnosis was confirmed via an upper gastrointestinal contrast study. Returning the stomach into the abdominal cavity via air insufflation enabled easy insertion of the enteral tube. A feeding tube might help avoid emergency intervention in the neonatal period and prevent fatal conditions, such as acute gastric volvulus or perforation. Further accumulation of cases is needed to clarify the optimal choice of fundoplication of congenital hiatal hernia.
